# Comparative study of the transfection efficiency of commonly used viral vectors in rhesus monkey (*Macaca mulatta*) brains

**DOI:** 10.24272/j.issn.2095-8137.2017.015

**Published:** 2017-03-18

**Authors:** Shi-Hao Wu, Zhi-Xing Liao, Joshua D. Rizak, Na Zheng, Lin-Heng Zhang, Hen Tang, Xiao-Bin He, Yang Wu, Xia-Ping He, Mei-Feng Yang, Zheng-Hui Li, Dong-Dong Qin, Xin-Tian Hu

**Affiliations:** ^1^Key Laboratory of Animal Models and Human Disease Mechanisms of the Chinese Academy of Sciences & Yunnan Province, Kunming Institute of Zoology, Chinese Academy of Sciences, Kunming Yunnan 650223, China; ^2^Nerve System Coding Discipline Group, Kunming College of Life Science, University of the Chinese Academy of Sciences, Kunming Yunnan 650000, China; ^3^Kunming Primate Research Center, Kunming Institute of Zoology, Chinese Academy of Sciences, Kunming Yunnan 650223, China; ^4^Department of Anatomy and Histology & Embryology, Faculty of Basic Medical Science, Kunming Medical University, Kunming Yunnan 650500, China; ^5^CAS Center for Excellence in Brain Science and Intelligence Technology, Chinese Academy of Sciences, Shanghai 200031, China; ^6^Center for Excellence in Brain Science, Wuhan Institute of Physics and Mathematics, Chinese Academy of Sciences, Wuhan Hubei 430071, China

**Keywords:** Recombinant adeno-associated virus, Lentivirus, Rhesus monkey, Central nervous system

## Abstract

Viral vector transfection systems are among the simplest of biological agents with the ability to transfer genes into the central nervous system. In brain research, a series of powerful and novel gene editing technologies are based on these systems. Although many viral vectors are used in rodents, their full application has been limited in non-human primates. To identify viral vectors that can stably and effectively express exogenous genes within non-human primates, eleven commonly used recombinant adeno-associated viral and lentiviral vectors, each carrying a gene to express green or red fluorescence, were injected into the parietal cortex of four rhesus monkeys. The expression of fluorescent cells was used to quantify transfection efficiency. Histological results revealed that recombinant adeno-associated viral vectors, especially the serotype 2/9 coupled with the cytomegalovirus, human synapsin Ⅰ, or Ca^2+^/calmodulin-dependent protein kinase Ⅱ promoters, and lentiviral vector coupled with the human ubiquitin C promoter, induced higher expression of fluorescent cells, representing high transfection efficiency. This is the first comparison of transfection efficiencies of different viral vectors carrying different promoters and serotypes in non-human primates (NHPs). These results can be used as an aid to select optimal vectors to transfer exogenous genes into the central nervous system of non-human primates.

## INTRODUCTION

The ability to manipulate the expression of genes in neurons using viral vectors allows for the transfer of exogenous genes into the central nervous system (CNS), thus providing a new approach to probe the neurological and mechanistic bases for brain functions and disorders (Costantini et al., 2000; Davidson & Breakefield, 2003). Recently, a range of novel and sophisticated transgenic tools have been used in CNS studies, including CRISPR/Cas9 and optogenetics technologies (Fenno et al., 2011; Ran et al., 2013). These gene editing technologies require viral vector transfection systems to transfer exogenous genes into various types of neurons, glias, and other cell types. To date, nine viral vector species have been used in CNS studies (Davidson & Breakefield, 2003). Among them, adeno-associated virus (AAV) and lentivirus vectors are the most advantageous due to their simplicity, low pathogenicity, and relative ease of production (Wong et al., 2006). 

AAV is a non-pathogenic member of the parvoviridae family belonging to the dependovirus genus, and induces weak host immune response (Bennett, 2003; Mingozzi & High, 2013; Nayak & Herzog, 2010; Xiao, 2003). This virus consists of a single-stranded genome and can deliver gene cassettes of approximately 4.7 kilobases (Carter & Samulski, 2000). AAV is a replication-defective virus and requires co-infection with a helper virus, such as adenovirus, to propagate, providing further safety for its application (Xiao, 2003). Moreover, AAV vectors can be purified to the highest titer in comparison to other viruses, which is advantageous because higher titers often result in higher transfection efficiencies (Davidson & Breakefield, 2003). In addition, AAV is a highly stable virus that is resistant to detergents, proteases, and organic solvents, and therefore is not affected by pH or temperature changes (Xiao, 2003). AAV also has many serotypes for transfecting different cell types (Burger et al., 2004; Schmidt et al., 2008; Xiao et al., 1999). For example, AAV1 is used to transfect skeletal muscle cells (Bish et al., 2008), AAV4 is used for transfecting ependymal cells (Davidson et al., 2000), and AAV8 is used for transfecting liver cells (Gao et al., 2002). Previous studies have shown that AAV1, 2, 5, 8, and 9 can transfect neurons and glia cells into various brain regions (Bartlett et al., 1998; Burger et al., 2004; Cearley & Wolfe, 2006; Gao et al., 2002; Towne et al., 2010), with their transfection efficiencies compared in this study.

It has been difficult to directly compare tropisms among various AAV serotypes because different Rep and Cap genes can lead to different tropisms (Burger et al., 2004; Taymans et al., 2007; Zincarelli et al., 2008). Therefore, to efficiently compare the transfection ability of certain serotypes in monkey brains, cross-packaged virions, such as AAV2/8 or AAV2/9, were utilized in this experiment (Rabinowitz et al., 2002). These virions were packaged with Rep genes from serotype 2 and Cap genes from another serotype. This allowed us to compare the effects of the Cap genes, while excluding the impacts of the Rep gene (Burger et al., 2004; Taymans et al., 2007; Zincarelli et al., 2008). As such, recombinant adeno-associated virus (rAAV) serotypes were chosen for this study, and included rAAV2/2, rAAV2/5, rAAV2/8, and rAAV2/9. 

Lentiviral vectors are derived from human immunodeficiency virus type 1 (HIV-1) and are often used to broaden the spectrum of transfection by containing a vesicular stomatitis virus G-protein (Cronin et al., 2005; Costantini et al., 2000; Lai & Brady, 2002; Naldini et al., 1996). Lentiviral vectors have a large genetic capacity (10 kb) and can integrate stably into the host cell genome of non-dividing cells such as neurons and in hematopoietic stem cells (Arhel et al., 2006; Lewis et al., 1992; Wiznerowicz & Trono, 2005). These features have helped achieve highly successful gene transfer because lentiviruses have the ability to transfect post-mitotic neurons in the CNS, which are generally considered refractory to infection (Cockrell & Kafri, 2007; Lewis et al., 1992; Miller et al., 1990; Wiznerowicz & Trono, 2005).

Researchers have shown growing interest in the application of new gene editing technologies in non-human primates (NHPs) as the differences between rodents and humans in brain size, structure, and function, as well as cell type and neural circuitry (Jennings et al., 2016), make brain disorder research on rodents less effective and often not directly applicable to humans (Okano et al., 2012). NHPs are considered ideal experimental animals for human disease modeling due to their high genetic similarity (Kirik et al., 2002). Moreover, NHPs are like humans in overall brain structure and social group living, thus providing a better reflection of the occurrence and development of human brain disorders (Shultz et al., 2011). Although lentiviral and AAV vectors are the most attractive genetic manipulation tools in developing transgenic models, their application has been primarily restricted to rodents (Aschauer et al., 2013; Weinberg et al., 2013), with many mature transgenic rodent models used in fundamental research and clinical trials (Elder et al., 2010; Kaplitt et al., 1994; Orth & Tabrizi, 2003; Ridet et al., 2006; Yang et al., 2013). However, similar transfection systems have been rarely used in NHPs. Since 2014, when the CRISPR/Cas9 with lentiviral vector system was first applied to NHPs (Niu et al., 2014), only a few transgenic monkeys have been developed (Liu et al., 2016;Niu et al., 2014; Niu et al., 2015; Zhang, 2017) and have not fully simulated the pathological symptoms of human disease, thereby limiting their widespread use. Different viruses, promoters, serotypes, and target brain regions affect the transfection efficacy of gene transfer (Scheyltjens et al., 2015). Therefore, another major hurdle in the application of AAV and lentiviruses to NHPs is the deficient knowledge on the identification of viral vectors that can lead to efficient and long-lasting expression in specific brain regions. Therefore, the full application of AAV and lentiviral vectors in NHPs remains a challenge (Watakabe et al., 2015). To date, most research has focused on the effects of different AAV serotypes in specific brain areas, such as the substantia nigra or corpus striatum (Markakis et al., 2010; Gerits et al., 2015; Watakabe et al., 2015). In the present study, we compared commonly used AAV and lentiviral vectors with different promoters and serotypes in the parietal cortex of rhesus monkeys to determine the optimal vector to deliver transgenes into the neocortex of NHPs. Eight commonly used AAV vectors and three lentiviral vectors with different promoters and serotypes were used. 

## MATERIALS AND METHODS

### Subjects

Four male adult rhesus monkeys (*Macaca mulatta*) weighing 5-9 kg and ranging in age from 10 to 15 years (mean±*SE*) were used as study subjects. The monkeys were housed in individual cages under typical conditions (temperature 21±2 ℃, 14/10 h light/dark cycle, light from 0700h to 2100h, humidity 60%) and fed with standard monkey chow and daily supplements of fruit (Yang et al., 2014). All animals were handled in accordance with the Kunming Institute of Zoology policies for use of laboratory animals under the National Care and Use of Animals guidelines approved by the Chinese National Animal Research Authority, in conformance with international guidelines for the ethical use of animals.

### Viral vectors

Details of the lentivirus and AAV viral vectors used in this study are listed in Table 1. Viruses ID1, ID2, ID4, ID10, ID11 were purchased from Obio Technology Co., Ltd. (Shanghai, China), viruses ID3 and ID7 were purchased from BrainVTA Technology Co., Ltd. (Wuhan, China), ID5 and ID8 from our lab and all other vectors were provided by partner laboratories (ID6 from Dr. Li-Ping Wang at the Shenzhen Institute of Advanced Technology, Chinese Academy of Sciences, Shenzhen, China, and ID9 from Dr. Zi-Long Qiu at the Shanghai Institutes for Biological Sciences, Chinese Academy of Sciences, Shanghai, China). All viruses carried the enhanced green fluorescent protein (eGFP) or mCherry gene sequence. The viruses were diluted in sterile phosphate buffered saline (PBS) to match their titers. The titers for the AAVs were 3.2×10^12^-4.3×10^12^ vg/mL and for the lentiviruses were 4.6×10^8^ TU/mL. Expressions of the eGFP or mCherry proteins by neurons were used to indicate transfection efficiency. 

**Table 1:** Details of the 11 viral vectors used

Virus ID	Exogenous gene	Promoter	Type	Serotype
ID1	eGFP	CMV	rAAV	AAV2/9
ID2	eGFP	CMV	rAAV	AAV2/2
ID3	mCherry	hSyn	rAAV	AAV2/9
ID4	eGFP	hSyn	rAAV	AAV2/8
ID5	eGFP	CAG	rAAV	AAV2/8
ID6	eGFP	CaMKII	rAAV	AAV2/5
ID7	mCherry	CaMKII	rAAV	AAV2/9
ID8	eGFP	hSyn	rAAV	AAV2/5
ID9	eGFP	UbC	Lentivirus	None
ID10	eGFP	hSyn	Lentivirus	None
ID11	eGFP	CMV	Lentivirus	None

Abbreviations: hybrid CMV-chicken β-actin (CAG), cytomegalovirus (CMV), human ubiquitin C (UbC), human synapsin Ⅰ (hSyn), Ca^2+^/calmodulin-dependent protein kinase Ⅱ (CaMKII) (Alexopoulou et al., 2008; Gruh et al., 2008; Kügler et al., 2003; Mayford et al., 1996; Schorpp et al., 1996; Wilhelm et al., 2011).

### Surgery

**Figure 1:**
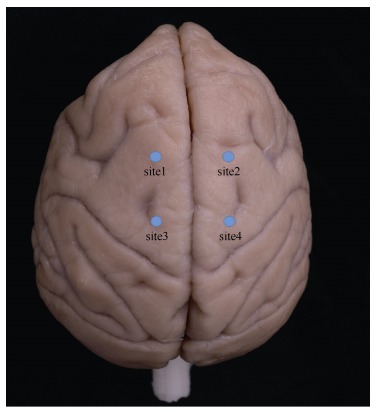
Distribution map of the injection sites on the rhesus monkey brain

Prior to surgery, all monkeys were anesthetized with atropine (0.03-0.05 mg/kg, i.m.), then injected with ketamine (10 mg/kg, i.m.) and pentobarbital (30 mg/kg, i.m.). All surgical procedures were conducted under strict aseptic conditions. As a prophylactic antibiotic treatment, cephalosporin was injected for three consecutive days after surgery (25 mg/kg/day, i.m., once a day). Monkeys were placed in a RWD stereotaxic frame (Product Model: 68902, Shenzhen, China) and two pieces of bone from the skull above the parietal cortex (~2 cm×1 cm each) were removed. The dura was carefully stripped to expose the brain parenchyma and the vectors were then infused through a 31-gauge Hamilton syringe placed in a syringe pump (WPI Apparatus, Sarasota, USA) attached to a stereotaxic instrument. Vectors were infused bilaterally into the brain using the following stereotaxic coordinates: (Injection Site 1 and Site 2): flat skull-anterior-posterior +25.05 mm, medial-lateral ±8 mm, and dorsal-ventral 3 mm from parenchyma surface, interaural=0; (Injection Site 3 and Site 4): anterior-posterior +10.05 mm, medial-lateral ±8 mm, and dorsal-ventral 3 mm from parenchyma surface, interaural=0. A map of the injection sites is shown in Figure 1. The vectors (5 μL) were infused at a rate of 400 nL/min. Following infusion, the needle was left in place for 8 min before being slowly retracted from the brain. After infusion, the dura was sutured and the bones were put back. 

The four monkeys were injected with one vector per injection site. Viruses (ID1, 2, and 4) were injected into Monkey 1 (Site 1, Site 2, and Site 3), viruses (ID5, 6, 8, and 9) were injected into Monkey 2, viruses (ID10 and 11) were injected into Monkey 3 (Site 1 and Site 3), and viruses (ID3 and 7) were injected into Monkey 4(Site 1 and Site 3). Blank sites were injected with other viruses (data not shown). The distances between the injection sites were greater than 15 mm to prevent diffusion of different viruses into one another. 

### Histology and microscopy

Six weeks after the viral vectors were injected, the monkeys were anesthetized with pentobarbital and transcardially perfused with 1 000 mL of PBS and 500 mL of 4% paraformaldehyde. The brains were then removed from the skull and fixed with 4% paraformaldehyde for 5d. Afterwards, the brains were equilibrated in 20%-30% sucrose until they sank. The frozen brains were sliced (40 μm) in a Leica Cryostat (CM1850 UV; Solms, Germany). The fluorescent signals were strong enough to be detected without enhancement by immunodetection, and were observed by inverted fluorescence microscopy (Leica DMI 6000B; Camera: Leica DFC 290; Optical master: Leica LTR6000, Wetzlar, Germany).

### Statistical analysis

For measurement of the eGFP or mCherry positive cells, a circle was drawn to cover the center of the positive cells. At the center of the circle a single optical section was captured using a 10× objective lens (Leica DMI 6000B; Camera: Leica DFC 290; Optical master: Leica LTR6000, Wetzlar, Germany). A region of interest (ROI, about 1.1 mm×1.1mm) was selected from the image. Morphologically intact neurons were counted. Fluorescence-positive cells were averaged by counting four slices from each viral injection site. Statistical relationships were determined using GraphPad (version 5.01) with one-way analysis of variance with Bonferroni multiple comparison tests. The level of significance was set at *P*<0.05, and all data were presented as means±*SE*.

## RESULTS

In the present study, eight AAV and three lentiviral vectors were tested in the rhesus monkey brain. Among these viral vectors, four AAV vectors and one lentiviral vector successfully transfected the brain.

**Figure 2:**
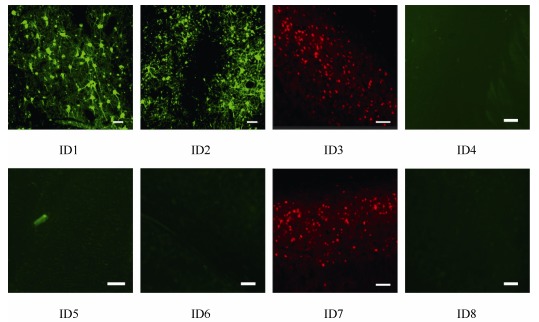
Expression of eGFP (ID1, ID2, ID4, ID5, ID6 and ID8) or mCherry (ID3 and ID7) using different viral vectors injected into the parietal cortex of the rhesus monkeys

**Figure 3:**
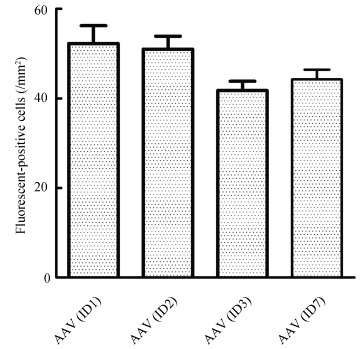
Comparison of transfection efficiencies of different viral vectors in the parietal cortex of rhesus monkeys

Of the eight AAV vectors, AAV2/9 (vector ID1) and AAV2/2 (vector ID2) carrying the CMV promoter, as well as AAV2/9 carrying the hSyn (vector ID3) and CaMKII (vector ID7) promoters, successfully transfected the brain (Figure 2). It is worth noting that AAV2/9 with the hSyn and CaMKII promoter was successfully transfected, whereas AAV2/5 and AAV2/8 with the same promoters failed. Additionally, AAV2/9 with the CMV promoter was also successfully transfected. The number of transfected neurons and the cellular morphology, as judged by the fluorescence signals, were compared for AAV2/9 coupled with three different promoters (ID1, ID3, and ID7). The neurons transfected with AAV2/9 vectors (ID1, ID3, and ID7) displayed a strong fluorescence signal in distal and thin dendrites (Figure 2). Statistical analysis revealed that the numbers of fluorescent positive neurons transfected with AAV2/9 with different promoters were not significantly different from each other (Figure 3). Thus, we inferred that the AAV2/9 serotype might be an ideal candidate serotype, and could be coupled with many commonly used promoters, resulting in high transfection efficacy in NHPs.

Details of the AAV vectors are reported in Table 2. The eGFP or mCherry expression profiles of the AAV vectors are shown in Figure 2. 

**Table 2:** Details of AAV vectors, including promoters and serotypes, and their expression

Virus ID	Promoter	Serotype	Expressed
ID1	CMV.	AAV2/9	Yes
ID2	CMV.	AAV2/2	Yes
ID3	hSyn	AAV2/9	Yse
ID4	hSyn	AAV2/8	No
ID5	CAG	AAV2/8	No
ID6	CaMKII	AAV2/5	No
ID7	CaMKII	AAV2/9	Yes
ID8	hSyn	AAV2/5	No

**Figure 4:**
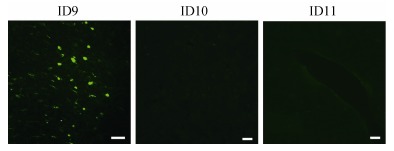
Expression of eGFP six weeks after intracranial injection of lentiviral vectors

Of the tested lentiviral vectors, only one with the UbC promoter was successfully transfected into the monkey brain. This suggests that the UbC promoter might have a strong effect at enhancing lentivirus expression in NHPs. Details of the lentiviral vector transfections are reported in Table 3. The eGFP expression profiles of the three lentiviruses are shown in Figure 4.

**Table 3:** Lentiviral vector expression in the rhesus monkey brain (vector ID7 successfully expressed eGFP in the monkeys)

Virus ID	Promoter	Type	Expressed
ID9	UbC	Lentiviruses	Yes
ID10	hSyn	Lentiviruses	No
ID11	CMV	Lentiviruses	No

## DISCUSSION

NHPs are important experimental animals in the development of genetic animal models of human diseases. The potential application of viral technologies to develop genetic models makes monkeys ideal candidates for therapeutics research (Jennings et al., 2016; Samaranch et al., 2012). However, only a few studies have compared the transfection efficiencies of different viral vectors in NHPs. As yet, most research has focused on the comparison of different AAV serotypes (Davidoff et al., 2005; Dodiya et al., 2010; Gerits et al., 2015; Lawlor et al., 2009; Watakabe et al., 2015), with no studies comparing the efficiency of AAV and lentivirus transfection with commonly used promoters and serotypes in NHPs. The goal of the current research was to identify the best candidates from the most commonly used AAV and lentiviral vectors to improve the success rate of NHP genetic models. Recombinant AAV and lentiviral vectors containing fluorescent protein genes were injected to the parietal cortex of four rhesus monkeys, with the expression of fluorescent cells then used to quantify transfection efficiency. We found that the CMV promoter within AAV2/2 and the UbC promoter within lentivirus resulted in higher expression of fluorescent cells. The AAV2/9 vector coupled with CMV, hSyn, or CaMKII promoters led to similar and high expression of fluorescent cells. 

Our results demonstrated that AAV2/9 exhibited higher transfection efficiency than that of AAV2/5 or AAV2/8 in the CNS of rhesus monkeys, although they carried the same promoters. Furthermore, AAV coupled with the CMV or hSyn promoters showed successful transfection, but the lentivirus with these two promoters failed. Considering that AAV and lentivirus belong to different virus genera, the production processes and titration methods were different. Therefore, the transfection efficacies between the AAV and lentivirus were not compared in the present study. 

Selecting a suitable promoter is a key factor in achieving successful transfection (Davidson & Breakefield, 2003). In this research, CMV, hSyn, CaMKIIα, CAG, and UbC were evaluated as alternative promoters. The CMV promoter was found to have higher transcription activity in AAV. It is worth noting that although the CMV promoter led to higher transcription activities in neurons, it has also been found to transcribe genes in other cell types, such as glial cells (Dittgen et al., 2004; Miyoshi et al., 1998; Zufferey et al., 1997). Therefore, the application of the CMV promoter is limited to research that solely focuses on neurons. The hSyn and CaMKIIα promoters are known to activate only neurons (Hioki et al., 2007), and had higher transcription activities with AAV2/9 than with AAV2/5 or AAV2/8. In addition, AAV2/9 coupled with the CMV promoter also resulted in high expression, suggesting that AAV2/9 can be used in neuron-type specific or non-specific transfection, thus making it an ideal candidate vector in NHP brain research. Concerning the lentivirus vectors, only the UbC promoter resulted in successful transfection. The UbC promoter regulates the expression of transgenes in a wide range of cells, as suggested by the expression of ubiquitin proteins in all eukaryotic cells, and is also able to confer high protein expression in neurons, whereas other promoters, including CMV, exhibit reduced activity (Schorpp et al., 1996; Wilhelm et al., 2011).

In conclusion, this study identified optimal viral vectors for gene transfer in NHP brains. Eight recombinant AAV and three lentiviral vectors, which are widely used in rodent brain research, were injected into the parietal cortex of rhesus monkeys, with the expression of fluorescent cells then used to quantify transfection efficiency. Six weeks after the injection, one lentiviral vector and four AAV vectors were found to have successfully induced the expression of fluorescent cells in the monkey brains, representing successful transfection. This study also evaluated the specific promoters and serotypes contained within the tested vectors and found that AAV2/2 coupled with the CMV promoter and lentivirus coupled with the UbC promotor resulted in successful transfection. Further analysis found that AAV2/9 coupled with different promoters led to high and stable transcription activities in the brains of rhesus monkeys, suggesting that AAV2/9-based vectors containing different promoters (CMV, hSyn, and CaMKIIα) might be ideal for NHP genetic modeling and brain research.

## ACKNOWLEDGEMENTS

We would like to thank Li-Ping Wang's lab at the Shenzhen Institute of Advanced Technology, Chinese Academy of Sciences, and Zi-Long Qiu's lab at the Shanghai Institutes for Biological Sciences, Chinese Academy of Sciences, for donating viral sources for this research.
